# Identification and Safety Assessment of *Enterococcus casseliflavus* KB1733 Isolated from Traditional Japanese Pickle Based on Whole-Genome Sequencing Analysis and Preclinical Toxicity Studies

**DOI:** 10.3390/microorganisms12050953

**Published:** 2024-05-08

**Authors:** Shohei Satomi, Shingo Takahashi, Takuro Inoue, Makoto Taniguchi, Mai Sugi, Masakatsu Natsume, Shigenori Suzuki

**Affiliations:** 1 Diet and Well-Being Research Institute, KAGOME Co., Ltd., 17 Nishitomiyama, Nasushiobara 329-2762, Tochigi, Japan; shingo_takahashi@kagome.co.jp (S.T.); takuro_inoue@kagome.co.jp (T.I.); shigenori_suzuki@kagome.co.jp (S.S.); 2 Genome Lead Co., Ltd., 2-3-35 Tokiwa-chou, Takamatsu 760-0054, Kagawa, Japan; makoto@tani8020.jp; 3 BioSafety Research Center Inc., 582-2 Shioshinden, Iwata 437-1213, Shizuoka, Japan; m-sugi@anpyo.co.jp (M.S.); m-natsume@anpyo.co.jp (M.N.)

**Keywords:** *Enterococcus casseliflavus*, whole-genome sequencing, antibiotic resistance, virulence gene, cytotoxicity, mutagenicity

## Abstract

The present study involves the precise identification and safety evaluation of *Enterococcus casseliflavus* KB1733, previously identified using 16S rRNA analysis, through whole-genome sequencing, phenotypic analysis, and preclinical toxicity studies. Analyses based on the genome sequencing data confirm the identity of KB1733 as *E. casseliflavus* and show that the genes related to vancomycin resistance are only present on the chromosome, while no virulence factor genes are present on the chromosome or plasmid. Phenotypic analyses of antibiotic resistance and hemolytic activity also indicated no safety concerns. A bacterial reverse mutation test showed there was no increase in revertant colonies of heat-killed KB1733. An acute toxicity test employing heat-killed KB1733 at a dose of 2000 mg/kg body weight in rats resulted in no deaths and no weight gain or other abnormalities in the general condition of the animals, with renal depression foci and renal cysts only occurring at the same frequency as in the control. Taking the background data into consideration, the effects on the kidneys observed in the current study were not caused by KB1733. Our findings suggest that KB1733 is non-pathogenic to humans/animals, although further studies involving repeated oral toxicity tests and/or clinical tests are required.

## 1. Introduction

Lactic acid bacteria (LAB) have historically had important applications in fermentation and food preservation, in addition to their health benefits being well recognized. According to the definition suggested by the Food and Agriculture Organization (FAO)/World Health Organization (WHO), health-promoting LAB are designated as probiotics and are considered non-pathogenic live microorganisms that confer health benefits to the host when administered in adequate amounts [1]. Non-viable LAB have also recently attracted interest as potential probiotics [2,3]. Most LAB employed as probiotics belong to the genera *Lactobacillus* and *Enterococcus* [4]. In our laboratory, we isolated heat-killed *Enterococcus casseliflavus* KB1733, which was previously identified as *E. casseliflavus* based on 16S rRNA sequence analysis and reported to induce significantly higher levels of interferon lambda 1 (IFN-λ1) than *E. casseliflavus* JCM 8723^T^ in intestinal epithelial cells [5]. Members of the IFN-λ family have been reported to provide antiviral protection at anatomical barriers such as the respiratory, hepatic, and gastrointestinal mucosas [6]; therefore, oral intake of *E. casseliflavus* KB1733 is expected to provide protection against enterovirus via IFN-λ molecules. It has thus been proposed that heat-killed *E. casseliflavus* KB1733 is a unique probiotic that may be potentially applied in functional foods and beverages as a preventive against infectious diseases [5].

Members of the genus *Enterococcus* are ubiquitous in nature and constitute normal inhabitants of the gastrointestinal tract of humans and animals, with some members being investigated for potential as probiotics [7,8]. However, some enterococci are known to be opportunistic pathogens and are prevalent causes of nosocomial infections [9]. To date, *Enterococcus* species have not obtained GRAS (generally recognized as safe) status or been recommended for the QPS (qualified presumption of safety) list of the European Food Safety Authority (EFSA) [10,11]. Therefore, the safety properties of enterococci need to be further investigated prior to their proposed use in the food industry.

Safety assessment with respect to antibiotic resistance, virulence factors, and animal experimentation is an important phase in the choice of enterococci as potential probiotics [12,13]. The pathogenicity of enterococci is associated with their complement of virulence factors and their resistance to a broad range of antibiotics, both of which are determined by the presence of intrinsic and/or acquired genes located on either the chromosome or plasmids. In particular, plasmids harboring resistance genes present a risk of horizontal gene transfer between humans and animals [12], which results in an increased incidence of enterococcal strains resistant to multiple classes of antibiotics through genetic mutations, thereby promoting their survival [9]. Some of the virulence factors of enterococci are sporadically detected in dairy isolates, such as the aggregation substance *asa1*, gelatinase (*gelE*), collagen adhesin (*ace*), enterococcal surface protein (*esp*), and cytolysin (*cylA*) [14]. These antibiotic resistance and virulence factors enable enterococci to invade the host and cause nosocomial infections [15]. In addition to the characteristics of the strain itself, the influence on the host should be investigated, such as geno- and acute oral toxicity. This is of particular importance prior to application in humans because of growing concern regarding the safety of probiotic components and their possible harmful effects on host DNA [16,17].

To date, the safety characteristics of *E. casseliflavus* for human consumption have not been fully elucidated. Whole-genome sequencing provides a comprehensive understanding of the genome of microorganisms, enabling species identification as well as the profiling of antibiotic resistance genes and virulence factors. Phenotypic and preclinical analyses can also provide useful information to evaluate any potential adverse effects of a microorganism on the host. Thus, the aim of this study was to assess the safety status of a potential immuno-stimulatory strain, *E. casseliflavus* KB1733, based on whole-genome sequencing analysis and preclinical toxicity studies.

## 2. Materials and Methods

### 2.1. Whole-Genome Sequencing Analysis

#### 2.1.1. Bacterial Strains and Growth Conditions

*E. casseliflavus* KB1733 was provided by the Diet and Well-being Research Institute, KAGOME Co., Ltd. (Nasushiobara, Tochigi, Japan). It was isolated from a traditional Japanese pickle, *wasabina-duke*, and was stored at −80 °C in freeze-dried form. *E. casseliflavus* JCM 8723^T^ (which is identical to *E. casseliflavus* ATCC 25788), selected as a control strain, was provided by RIKEN BRC through the National BioResource Project of the MEXT/AMED, Japan. Each bacterium was inoculated with 1% (*v*/*v*) of a thawing glycerol stock in 10 mL de Man, Rogosa, and Sharpe (MRS) broth (Difco, Detroit, MI, USA) and incubated for 20 h at 30 °C.

#### 2.1.2. DNA Preparation, Genome Sequencing, and De Novo Hybrid Assembly

We prepared genomic DNA from late-logarithmic phase *E. casseliflavus* KB1733 and *E. casseliflavus* JCM 8723^T^ by lysing the cell wall using an enzyme, based on a previously reported protocol [18]. Briefly, the cells were digested using lysozyme and achromopeptidase (FUJIFILM Wako Pure Chemical, Osaka, Japan) at 37 °C and then treated with SDS and proteinase K (TaKaRa, Shiga, Japan) at 57 °C. Furthermore, RNA was degraded using RNase (Nippon Gene, Tokyo, Japan). After the extraction of the lysate with chloroform phenol, the upper layer was precipitated using isopropanol, and the precipitate was eluted using TE buffer.

The *E. casseliflavus* KB1733 and *E. casseliflavus* JCM 8723^T^ genomes were both sequenced through hybrid assembly combining long-read sequencing using GridION X5 (Oxford Nanopore Technologies, Oxford, UK) and short-read sequencing using MiSeq (Illumina, San Diego, CA, USA).

For long-read sequencing, 1000 ng of genomic DNA was treated with Short Read Eliminator XS (Circulomics, Baltimore, MD, USA) to remove low-molecular-weight DNA of approximately 10 K base pairs (bp) or less. The Ligation Sequencing Kit SQK LSK10.9 (Oxford Nanopore Technologies) was used to prepare a library for nanopore sequencing from 600 ng of genomic DNA after removing low-molecular-weight DNA. Sequencing was performed using a MinION flow cell FLO-MIN106 R9.41revD (Oxford Nanopore Technologies).

For short-read sequencing, 500 ng of genomic DNA was prepared into a library according to the Nextera DNA Flex Library Prep Kit (Illumina) protocol. Libraries were quantified using digital PCR and accurately adjusted to 4 nM. The 4 nM library was denatured using 0.2 N NaOH to a final library concentration of 8 pM. PhiX was added to a final concentration of 15%, and 155 bp × 2 sequencing was performed using MiSeq.

The de novo hybrid assembly of long MinION reads and short Illumina reads was performed using Unicycler version 0.4.3 [19] after first removing low-quality reads (for short Illumina reads ≤ Quality score [Q] 30, and for long MinION reads ≤ Q10), excessively short reads (for short Illumina reads ≤ 10 base-pair [bp], and for long MinION reads ≤ 1000 bp), and Illumina adaptor sequences. The scaffolds after assembly were visualized using Bandage (a Bioinformatics Application for Navigating De Novo Assembly Graphs Easily) at https://rrwick.github.io/Bandage/ (accessed on 8 December 2020) [20]. We screened the quality- and adapter-trimmed data for contaminants using taxon-annotated GC-coverage plots (TAGC or blobplots) and the blobtools package (version 1.0) [21,22]. A circular representation of the complete genome sequence was visualized using the GView server (version 1.7) [23].

#### 2.1.3. Comparative Genome Analysis

In silico DNA–DNA hybridization was assessed using the average nucleotide identity (ANI) and the genome-to-genome distance calculator (GGDC). Both the ANI and the GGDC of *E. casseliflavus* KB1733 were compared with those of *E. casseliflavus* JCM 8723^T^. The ANI was determined using a previously described method and calculated using server-based software available at http://enve-omics.ce.gatech.edu/ani/ (accessed on 12 April 2023) with the following parameters: minimum length 700 bp, minimum identity 70%, minimum alignment 50%, BLAST window size 1000 bp, and step size of 200 bp. The server-based GGDC (3.0) software at https://ggdc.dsmz.de/distcalc2.php (accessed on 12 April 2023) was used under the recommended protocol, Formula 2.

All *E. casseliflavus* KB1733 genome sequence data were deposited in the DDBJ/GenBank/EMBL database at https://ddbj.nig.ac.jp/arsa/ (accessed on 6 April 2024) under the accession numbers AP031371 and AP031372.

### 2.2. Antibiotic Resistance and Virulence Factors

#### 2.2.1. Bacterial Strains and Growth Conditions

*E. casseliflavus* strains KB1733 and JCM 8723^T^ were evaluated in this study. *Enterococcus faecalis* JCM 7783 (which is identical to *E. faecalis* ATCC 29212) and *Staphylococcus aureus* JCM 2874 (which is identical to *S. aureus* ATCC 29213) were provided by the RIKEN BRC through the National BioResource Project of the MEXT/AMED, Japan, and were used as control strains. The four strains used in this study were stored in glycerol stock vials at −80 °C until use.

Mueller–Hinton (MH) broth (Becton Dickinson, Franklin Lakes, NJ, USA, product number: 275730) was used for the inoculation and passaging of each strain in culture. Each strain was inoculated with 1% (*v*/*v*) of a thawing glycerol stock in 10 mL MH liquid medium and then incubated for 20 h at 37 °C. After incubation, 100 µL of the culture was inoculated into 10 mL of fresh MH liquid medium and then incubated for 24 h at 37 °C. The culture medium was diluted to McFarland 1 (OD_600_ = 0.257) in fresh MH liquid medium, and the minimum inhibitory concentration (MIC) and hemolytic activity were determined.

#### 2.2.2. Antibiotic Resistance

We investigated the antibiotic resistance of *E. casseliflavus* KB1733 both genetically and phenotypically. Antibiotic resistance-related genes were confirmed using ResFinder (version 4.1). Horizontal gene transfers related to antibiotics resistance genes were confirmed using Alien Hunter (version 1.1.0).

The antibiotic resistance of target strains was assessed using ETEST^®^ (bioMerieux, Lyon, France) according to the manufacturer’s instructions. The MICs of ampicillin (AM) (# 412253), chloramphenicol (CL) (# 412309), ciprofloxacin (CI) (# 412311), clindamycin (CM) (# 412315), erythromycin (EM) (# 412334), gentamicin (GM) (# 412368), kanamycin (KM) (# 412382), linezolid (LZ) (# 412396), rifampicin (RI) (# 412450), tetracycline (TC) (# 421471), and vancomycin (VA) (# 412488) were evaluated. A strain cultured in MH liquid medium was diluted to McFarland 1 (OD_600_ = 0.257) and plated on MH agar medium (Becton Dickinson, product number: 225250). The plates were incubated under anaerobic conditions for 48 h to accommodate strain growth. The MIC value was determined at the position where the circle of inhibition crosses the antibiotic strip. The MICs for *E. casseliflavus* KB1733 and *E. faecalis* JCM 7783 were compared with those of the references suggested by the FEEDAP [24] or the CLSI [25].

#### 2.2.3. Virulence Factors

We investigated the virulence factors of *E. casseliflavus* KB1733 both genetically and phenotypically. Virulence genes were confirmed using VirulenceFinder (version 2.0).

Furthermore, we investigated the hemolytic activity of *E. casseliflavus* KB1733 using a sheep-blood agar medium. The strain cultured in MH liquid medium was diluted to McFarland 1 (OD_600_ = 0.257), and 100 μL of the cultured medium was plated onto the sheep-blood agar medium (Nissui Pharmaceutical Co., Ltd., Tokyo, Japan, product number: 51001). Dishes were incubated for 48 h under anaerobic conditions. The formation of a clear zone around the colonies was taken as indicating β-hemolytic activity (true hemolysis). A brown or greenish color change in the medium around the colonies was considered as α-hemolytic activity. Unaltered strains were considered devoid of hemolytic activity (γ-hemolysis). Strains exhibiting β-hemolytic activity were considered virulent.

### 2.3. Bacterial Reverse Mutation Test (Ames Test)

#### 2.3.1. Guidelines Compliance

The Ames test was carried out by the BioSafety Research Center Inc. It was performed with reference to the ‘Guidelines for the Designation of Food Additives and Revision of Standards for Use of Food Additives’ (22 March 1996) [26] and ‘Guidance on Genotoxicity Testing and Data Interpretation for Pharmaceuticals Intended for Human Use’ (20 September 2012) [27].

#### 2.3.2. Tester Microorganisms, Negative Control, and Positive Control

Tester microorganisms used in the Ames test included *Salmonella typhimurium* strains TA100, TA1535, TA98, and TA1537 (provided by Dr Bruce N. Ames, University of California, Berkeley, CA, USA) and *Escherichia coli* WP2*uvrA* (provided by the National Institute of Technology and Evaluation, Chiba, Japan).

Water for injection was used as a negative control for all test microorganisms. The following compounds were employed as positive controls in studies conducted according to the preincubation method without metabolic activation: 2-(2-furyl)-3-(5-nitro-2-furyl) acrylamide (AF-2) (FUJIFILM Wako Pure Chemical, Lot No. CAH4001) at 0.01 µg/plate for TA100 and WP2*uvrA* and at 0.1 µg/plate for TA98, 0.5 µg/plate of sodium azide (NaN_3_) (FUJIFILM Wako Pure Chemical, Lot No. RSJ7863) for TA1535, and 80 µg/plate of 9-aminoacridine hydrochloride (9-AA) (Sigma-Aldrich, St. Louis, MO, USA, Lot No. BCBW4667) for TA1537. In assays conducted using the same method in the presence of metabolic activation, 2-aminoanthracene (2-AA) (FUJIFILM Wako Pure Chemical, Lot No. PTL0822) was used as the positive control at the following concentrations: 0.5 µg/plate for TA98, 1.0 µg/plate for TA100, 2.0 µg/plate for TA1535 and TA1537, and 10 µg/plate for WP2*uvrA*.

#### 2.3.3. Metabolic Activation

Microsomal fractions (S9) isolated from the livers of phenobarbital- and 5,6-benzoflavone-induced male Sprague–Dawley rats were used to confirm the mutagenicity of *E. casseliflavus* KB1733 under metabolically active conditions. An S9 mix was prepared by adding 1 mL of S9 (Lot No. 21052102) into 9 mL of coenzyme A (Lot No. A21051802) and consisted of 0.1 mL of S9, 8 µmol of MgCl_2_, 33 µmol of KCl, 5 µmol of glucose-6-phosphate, 4 µmol of NADPH, 4 µmol of NADH, and 100 µmol of sodium phosphate (pH 7.4) (per 1 mL of S9 mix).

#### 2.3.4. Sample Preparation

After *E. casseliflavus* KB1733 was incubated as described in Section 2.1.1, 4 mL of cell culture was inoculated into 36 mL of MRS medium and further incubated for 20 h at 30 °C. The cell cultures were washed with sterilized saline and water, followed by centrifugation at 4 °C and 2590× *g* for 10 min (himac22G, R10A2, Eppendorf Himac Technologies, Hitachinaka, Ibaraki, Japan). In accordance with a previous report [5], washed bacteria were autoclaved at 100 °C for 30 min (Iwaki, Tokyo, Japan) and freeze-dried for sample preparation.

#### 2.3.5. Experimental Procedure

To establish appropriate concentrations for the study and provide a preliminary evaluation of mutagenicity, a dose-finding (preliminary) study was conducted using the preincubation method.

The preliminary study was performed at final heat-killed *E. casseliflavus* KB1733 concentrations of 8.19, 20.5, 51.2, 128, 320, 800, 2000, and 5000 μg/plate in the absence and presence of S9 (two plates per concentration). All plates were macroscopically observed for the precipitation of the test compound at the start of treatment and at the time of colony counting. Growth inhibitory effects were examined using a stereoscopic microscope, and revertant colonies were counted using an automated colony analyzer. A response was considered positive if the mean number of revertant colonies was two times greater than the negative control and either a concentration-dependent relationship was observed or the effects were reproducible. Statistical analysis of the data was not conducted.

After the preliminary study, the main study was performed at final heat-killed *E. casseliflavus* KB1733 concentrations (prepared by serial dilution) of 156, 313, 625, 1250, 2500, and 5000 μg/plate in the absence and presence of S9 (two plates per concentration). The other procedures in the main study were the same as those used in the preliminary study.

### 2.4. Acute Oral Toxicity

#### 2.4.1. Ethical Approval and Guidelines

The acute oral toxicity study was conducted according to the following guidelines: Act on Welfare and Management of Animals, Standards Relating to the Care and Management of Laboratory Animals and Relief of Pain, and Guidance for Animal Testing.

This study was reviewed and approved by the Animal Experiment Committee of BioSafety Research Center Inc. prior to commencement (Approval No. 21-0137A). It was conducted in accordance with ‘Revisions of Guidelines for Single and Repeated Dose Toxicity Studies’.

#### 2.4.2. Animals and General Housing Conditions

Specific-pathogen-free Sprague–Dawley Crl:CD rats were purchased from Charles River Japan, Inc. (Kanagawa, Japan). In total, six male and six female 5.5-week-old rats were employed in the acute oral toxicity study to evaluate the effects of heat-killed *E. casseliflavus* KB1733 on sex differences in toxicity. Of these, five male and five female rats at 6 weeks of age were administered the test substance (body weight range at administration: 184–195 g for males and 147–156 g for females).

Two or three rats were housed in a plastic cage (W 34.5 × D 40.3 × H 17.7 cm) with floor coverings (ALPHA-dri™, Lot No. 07120, Shepherd Specialty Papers, Scituate, MA, USA). The rearing cages, bedding, and feeders were changed at least once a week, and the water bottles were changed at least once every three days. The animals were fed ad libitum on solid feed (CRF-1, Lot No. 210304, Oriental Yeast Co., Ltd., Tokyo, Japan) except when fasting. Tap water was fed ad libitum from a water bottle.

When the animals arrived on 22 September 2021, serial numbers (males: M1–M6, females: F1–F6) were assigned to each rat and were written on the tail using an oil-based felt-tip pen. An animal identification number card (ID card) with the animal number clearly marked was also posted in the rearing cage to identify the animals.

The quarantine period was defined as the period between the receipt of animals (22 September 2021) and the day before administration (27 September 2021) when their body weights were measured. The acclimation period was defined as the period between the receipt of animals and the day of administration (28 September 2021). During the quarantine and acclimation period, the general condition of the animals was observed once a day, and their body weights were measured at the time of receipt and before fasting on the day before administration. No abnormalities were observed in any of the animals.

#### 2.4.3. Feeding Intervention

All animals were fasted overnight from the evening of the day before administration. On the day of administration (28 September 2021), general condition observations and weight measurements were carried out, and five out of six animals of each sex were selected in number order from those that showed no abnormalities and were deemed suitable for the acute oral toxicity test. Animals not selected were treated as surplus. The animals were re-fed at 3 h post-administration.

The test substance was suspended in water for injection and orally administered to the selected M1–M5 and F1–F5 rats using a plastic syringe and a Teflon gastric sonde. As the dosing formulation, a 200 mg/mL suspension was prepared. Heat-killed and freeze-dried KB1733 powder (6.0 g, described in Section 2.3.4) was weighed, suspended in water for injection (Lot No. K1C72, Otsuka Pharmaceutical Factory, Inc., Tokushima, Japan), and diluted to a final volume of 30 mL. The administration volume was set at 1 mL of the test substance per 100 g of body weight, which was the individual body weight on the day of administration. The dose was set at 2000 mg/kg, which is the upper limit of the dose for general acute oral toxicity studies in rodents specified in the Organization for Economic Cooperation and Development (OECD) guidelines.

#### 2.4.4. Clinical Observations, Measurements, and Outcomes

The observation period was between the date of administration (28 September 2021) and the date of autopsy (12 October 2021). The general condition of the animals was observed once before and within 30 min after administration, and then once an hour thereafter until 4 h after administration. Observations were performed once a day for the 14 days following administration.

Animal weights were measured immediately before, 7 days after, and 14 days after administration of the test substance. Pathological examination consisted of the gross observation (necropsy) of various organs and tissues throughout the body.

At the end of the observation period (day 14 after administration), all animals were euthanized under isoflurane anesthesia by blood release. At necropsy, the animals’ body surfaces and spontaneous openings were observed, with various organs and tissues throughout the body examined, including the abdominal, thoracic, pelvic, and cranial cavities. The location, size, and coloration of all gross abnormalities were recorded. After the autopsy, the cadavers were discarded.

Outcomes in the current test were set as follows: mortality, general condition, weight gain, and pathological examination. With reference to the OECD guidelines, individual animal data were provided; therefore, no control group was established, and no statistical analysis was carried out.

## 3. Results and Discussion

### 3.1. Whole-Genome Sequencing Analysis

**Figure 1:**
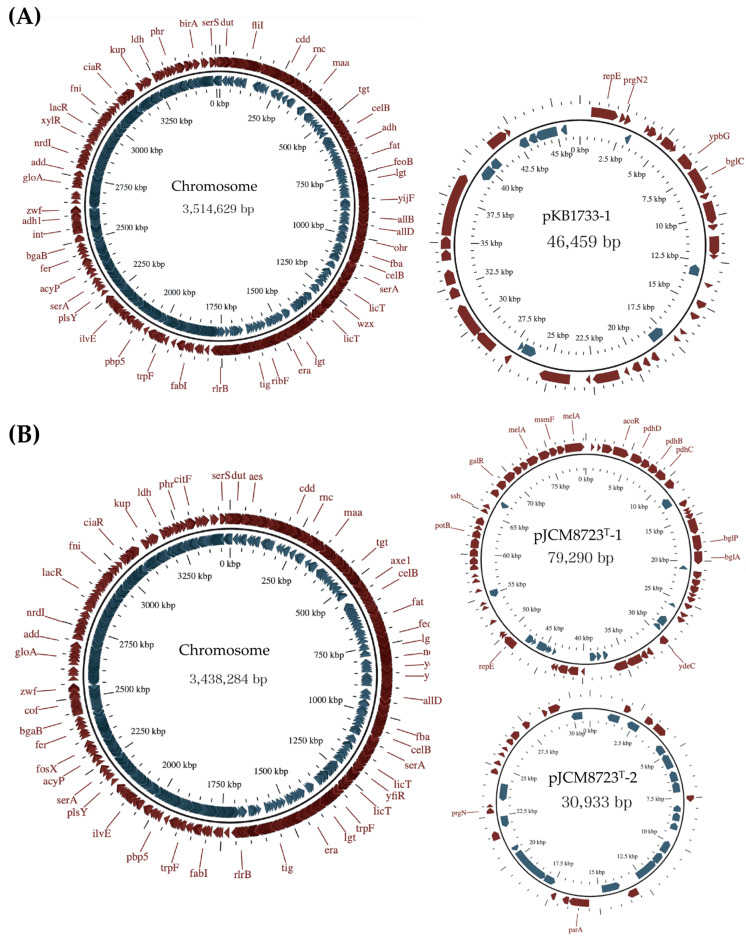
Circular representation of the genomes of *E. casseliflavus* KB1733 (**A**) and *E. casseliflavus* JCM 8723^T^ (**B**).

The genome of *E. casseliflavus* KB1733 comprises a circular chromosome of 3,514,629 bp and one plasmid of 46,459 bp. By contrast, the genome of *E. casseliflavus* JCM 8723^T^ comprises a circular chromosome of 3,438,284 bp and two plasmids of 79,290 bp and 30,933 bp (Figure 1). Regarding the *E. casseliflavus* KB1733 chromosome, the GC content is 42.8%, 15 rRNAs and 61 tRNAs were found, and no CRISPRs were present. In the *E. casseliflavus* KB1733 plasmid (pKB1733-1), the GC content is 34.4%, and rRNAs, tRNAs, and CRISPRs were not present. By contrast, for the *E. casseliflavus* JCM 8723^T^ chromosome, the GC content was 42.6%, 15 rRNAs and 60 tRNAs were found, and no CRISPRs were present. Regarding the *E. casseliflavus* JCM 8723^T^ plasmids, pJCM8723^T^-1 has a GC content of 35.4%, and there were no rRNAs, tRNAs, or CRISPRs found, and pJCM8723^T^-2 has a GC content of 31.2%, and rRNAs, tRNAs, and CRISPRs are not present (Table 1).

The results obtained using traditional methods to identify microorganisms, including morphological, physiological, and biochemical reactions, may differ greatly under different experimental conditions, leading to bias in the identification [28]. The values of tests based on whole-genome sequence data, ANI and GGDC, have been shown to correlate positively with those of DNA–DNA hybridization tests using genomic DNA extracts [29,30]. The ANI and GGDC of *E. casseliflavus* KB1733 tested against *E. casseliflavus* JCM 8723^T^ were 98.93% and 96.12%, respectively. The values for both the ANI and GGDC were clearly above the proposed criterion for bacterial species delineations (≥95% and ≥70%, respectively) [29,30]. The genus and species identification that resulted from using ANI and GGDC for *E. casseliflavus* KB1733 were the same as those previously identified using 16S rRNA sequence analysis (unpublished data).

### 3.2. Antibiotic Resistance

The results from ResFinder (4.1) indicate that the *vanC2* and *vanXY* genes of *E. casseliflavus* KB1733 are found on its chromosome, which is also the case with strain JCM 8723^T^ (Table 2). VA resistance is of great concern because VA-resistant enterococci are known to cause serious infections and diseases that cannot be treated using conventional antibiotic therapy [31,32]. There are six genes in enterococci known to cause VA resistance: *vanA*, *vanB*, *vanC*, *vanD*, *vanE*, and *vanG*. Among the genes related to VA resistance, only *vanA* and *vanB* are capable of both vertical and horizontal transfer and conferring high levels of resistance [33]. The *vanC* determinant induces low-level VA resistance and intrinsic sensitivity to teicoplanin [33], and *vanXY* is related to the *vanC* phenotype of glycopeptide resistance [34]. In general, the *vanA*, *vanB*, *vanD*, *vanE*, and *vanG* genes are considered acquired properties, while *vanC* is an intrinsic trait of motile enterococci. Furthermore, the results obtained from Alien Hunter (1.1.0) indicate that those contigs scoring lower than the threshold do not harbor resistance genes (*vanC* and *vanXY*) (Supplementary Table S1). In a previous review, genes or genomic regions with a score below or above the threshold are described as possibly atypical [35]. The results support the notion that *vanC* and *vanXY* are intrinsic traits in *E. casseliflavus* KB1733. Collectively, this indicates it is likely *E. casseliflavus* KB1733 is sensitive to VA and that its genes related to VA resistance would not transfer to other microorganisms.

To evaluate the antibiotic resistance traits of *E. casseliflavus* KB1733 and *E. casseliflavus* JCM 8723^T^, 11 antibiotics (including VA) were evaluated using the ETEST^®^ (Table 3). For the control strains, all antibiotic measurements are within the CLSI reference range [25], except for GM on *S. aureus* JCM 2874. For *E. casseliflavus* KB1733, the measured values of all antibiotics are equal to or lower than the standard values for the genus *Enterococcus* presented by the FEEDAP [24]. The values for *E. casseliflavus* JCM 8723^T^ are almost the same as those of *E. casseliflavus* KB1733. In addition, for *E. casseliflavus* KB1733, the measured values for all antibiotics are lower than or within the reference values for the genus *Enterococcus* published by the CLSI. The values for *E. casseliflavus* JCM 8723^T^ are also almost the same as those of *E. casseliflavus* KB1733. These results suggest that *E. casseliflavus* KB1733 is phenotypically and genetically sensitive to the antibiotics tested above.

### 3.3. Virulence Factors

**Figure 2:**
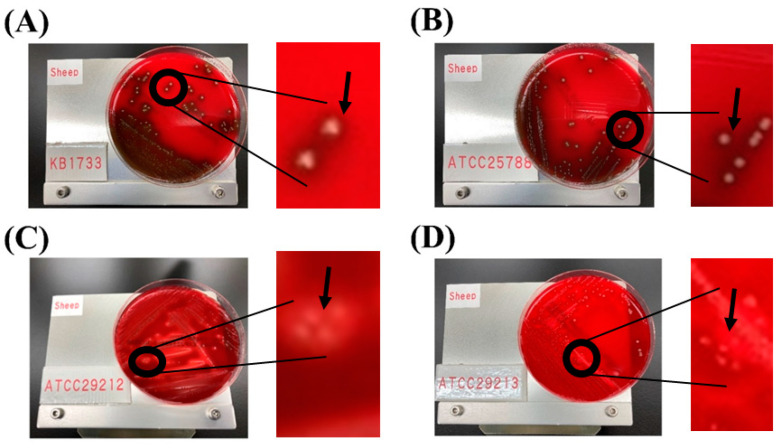
Hemolytic activity in *E. casseliflavus* KB1733 (**A**), *E. casseliflavus* JCM 8723^T^ (ATCC 25788) (**B**), *E. faecalis* JCM 7783 (ATCC 29212) (**C**), and *S. aureus* JCM 2874 (ATCC 29213) (**D**). Arrows indicate a brown zone or a clear zone around the colonies.

The results from VirulenceFinder (version 2.0) indicate that neither *E. casseliflavus* KB1733 nor *E. casseliflavus* JCM 8723^T^ (ATCC 25788) possess any virulence genes on their chromosomes or plasmids. Furthermore, after incubation on blood agar plates for 48 h, neither *E. casseliflavus* KB1733 nor *E. casseliflavus* JCM 8723^T^ exhibited any hemolytic effects, whereas the positive control strains, ATCC 29212 and ATCC 29213, showed clear hemolytic effects (Figure 2). The following enterococci virulence genes have been reported: gelatinase (*gelE*), enterococcal surface protein (*esp*), cytolysin (*cylA*), adhesion to collagen (*ace*), cell-wall adhesion (*efaA*), and cell adhesion (*agg*). Of these, cytolysin, which mediates bactericidal and hemolytic activity, is the major pathogenic factor of enterococci and is responsible for the main characteristic of enterococcal pathogenicity in clinical tests [36]. In the *Enterococcus* genus, hemolysin is one of the virulence traits [37]. However, *E. casseliflavus* KB1733 lacks these genes or virulence traits, indicating that it is a non-toxigenic strain.

### 3.4. Ames Test

All positive controls showed mutagenic activity, and the mean number of revertant colonies in the positive and the negative controls was within the range of historical background data. This confirms that the Ames test was properly conducted. Although precipitation of the test article at a concentration of heat-killed *E. casseliflavus* KB1733 over 2000 μg/plate was observed at the start of treatment or at the time of colony counting, no doubling of the number of revertant colonies for heat-killed *E. casseliflavus* KB1733 was recorded in either the preliminary study or the main study, regardless of the presence or absence of S9 mix (Table 4 and Table 5). The Ames test is an important non-clinical safety assay that can accurately predict the mutagenic effects of foods, chemicals, and plant extracts. In summary, our results suggest that heat-killed *E. casseliflavus* KB1733 is non-mutagenic.

### 3.5. Acute Oral Toxicity

The acute toxicity of heat-killed *E. casseliflavus* KB1733 was investigated at a dose of 2000 mg/kg weight in rats. No abnormalities in weight gains or losses were observed on day 7 and day 14 after administration of the test substances (Supplementary Table S2). No deaths or abnormalities in the general condition were observed in the 2 weeks between the date of administration and autopsy. Pathological examination revealed renal depressed foci in two out of five males (animal no. M2 and M3) and one out of five females (animal no. F3), and renal cysts were found in one out of five males (animal no. M2).

Historical background data on acute toxicity studies conducted at the BioSafety Research Center Inc. over a 5-year period (2011–2016) show that renal cysts occurred in up to 20% of both males and females, and renal depressed foci occurred in up to 40% of males and 60% of females. In the current study, the frequency of cysts was recorded as 20% for males and 0% for females, and the frequency of depressed foci was 40% for males and 20% for females, which is similar to the maximum frequency of occurrence in the historical data. This indicates that the effect on the kidneys observed in the current study is not caused by heat-killed *E. casseliflavus* KB1733. In future studies, the non-toxigenic nature of strain KB1733 should be confirmed on the basis of repeated, long-term administration.

Based on the above findings, and under the conditions of the study, the lethal dose of heat-killed *E. casseliflavus* KB1733 after a single oral administration to rats is considered to be >2000 mg/kg for both males and females.

## 4. Conclusions

Whole-genome sequencing analysis was used to confirm the identity of strain KB1733 as *E. casseliflavus*, the absence of virulence factor genes, and the untransferable nature of the antibiotic resistance genes due to the absence of plasmids. The results of phenotypic analyses correlated with the results of the complete genome analysis, the Ames test, and the acute oral toxicity test, all of which confirmed the safety profile of *E. casseliflavus* KB1733. The present study indicates that strain *E. casseliflavus* KB1733 is likely non-pathogenic to humans/animals and can be considered a potential probiotic candidate, although further studies involving repeated oral toxicity tests and/or clinical tests are needed.

## Figures and Tables

**Table 1:** General features of the two strains of *E. casseliflavus* ^1^.

	KB1733		JCM 8723^T^		
	Chromosome	pKB1733-1	Chromosome	pJCM8723^T^-1	pJCM8723^T^-2
Size (bp)	3,514,629	46,459	3,438,284	79,290	30,933
GC content (%)	42.8	34.4	42.6	35.4	31.2
Number of CDSs	3318	47	3277	78	38
Average protein length	309.4	238.8	304.4	267.4	187.5
Number of rRNAs	15	0	15	0	0
Number of tRNAs	61	0	60	0	0
Number of CRISPRs	0	0	0	0	0
Average read depth	59.113	81.5884	83.1	117.8	146.1

^1^ Abbreviations: bp, base pair; CDS, coding sequence; CRISPRs, clustered regularly interspaced short palindromic repeats; KB1733, *Enterococcus casseliflavus* KB1733; JCM 8723^T^, *Enterococcus casseliflavus* JCM 8723^T^ (ATCC 25788); pKB1733-1, one plasmid of KB1733; pJCM8723^T^-1, one plasmid of JCM8723^T^; pJCM8723^T^-2, the other plasmid of JCM8723^T^.

**Table 2:** Antibiotic resistance genes of *E. casseliflavus* KB1733 and JCM 8723^T 1^.

	Resistance Gene	Identity (%)	Contig	Position in Contig
KB1733	*vanC2*	100	Chr	2,624,186–2,625,238
	*vanXY*	99.3	Chr	2,625,235–2,625,807
JCM 8723^T^	*vanC2*	100	Chr	2,539,000–2,540,052
	*vanXY*	99.3	Chr	2,540,049–2,540,621

^1^ Abbreviations: Chr, chromosome; KB1733, *Enterococcus casseliflavus* KB1733; JCM 8723^T^, *Enterococcus casseliflavus* JCM 8723^T^ (ATCC 25788).

**Table 3:** MIC values of antibacterial drugs for the four test strains ^1^.

	Test Strains			Control Strains			
	KB1733	JCM 8723^T^	*Enterococcus*	JCM 7783		JCM 2874	
			Reference *		Reference ^†^		Reference ^†^
AM	0.18	0.25	2	0.5	0.5–2	0.5	0.5–2
CL	2.5	3	16	4	4–16	5	2–16
CI	1.5	1.8	NR	0.4	0.25–2	0.15	0.12–0.5
CM	2.5	2	4	12	4–16	0.08	0.06–0.25
EM	4	3	4	4	1–4	0.5	0.25–1
GM	3	2	32	5	4–16	2	0.12–1
KM	32	16	1024	16	16–64	3.5	1–4
LZ	1.5	1	NR	1.5	1–4	1.5	1–4
RI	0.75	0.5	NR	0.75	0.5–4	0.004	0.004–0.015
TC	0.25	0.19	4	12	8–32	0.125	0.12–1
VA	1	2	4	2	1–4	0.75	0.5–2

^1^ Abbreviations: AM, ampicillin; CL, chloramphenicol; CI, ciprofloxacin; CM, clindamycin; EM, erythromycin; GM, gentamicin; KM, kanamycin; LZ, linezolid; RI, rifampicin; TC, tetracycline; VA, vancomycin; KB1733, *Enterococcus casseliflavus* KB1733; JCM 8723^T^, *Enterococcus casseliflavus* JCM 8723^T^ (ATCC 25788); JCM 7783, *Enterococcus faecalis* JCM 7783 (ATCC 29212); JCM 2874, *Staphylococcus aureus* JCM 2874 (ATCC 29213); NR, not reported. * Suggested by the FEEDAP. ^†^ Suggested by the CLSI.

**Table 4:** Results of a dose-finding study for the bacterial reverse mutation test of *Enterococcus casseliflavus* KB1733 ^1^.

Dose (μg/Plate)	Mean Revertant Colonies per Plate									
	*Escherichia coli*		*Salmonella typhimurium*							
	WP2*uvrA*		TA100		TA1535		TA98		TA1537	
	−S9	+S9	−S9	+S9	−S9	+S9	−S9	+S9	−S9	+S9
Negative control *	22	19	116	121	10	12	20	35	12	16
8.19	23	15	121	124	13	9	28	35	11	14
20.5	21	21	132	110	8	6	24	31	13	11
51.2	20	25	111	106	6	11	28	31	6	19
128	22	22	99	117	7	13	29	27	6	16
320	31	16	113	125	16	15	26	30	8	7
800	17	26	114	123	10	5	23	30	6	9
2000^+^	23	27	110	124	10	9	21	29	5	12
5000^+^	22	19	115	120	8	11	28	39	9	10
Positive control ^†,‡^	113	869	558	892	566	332	640	350	258	149

^1^ Abbreviations: −S9, in absence of S9; +S9, in presence of S9; 2000^+^ and 5000^+^, precipitation of the test article was observed at 2000 and 5000 μg/plate, respectively. * Water for injection was used as a negative control. ^+^ Precipitation of the test article was confirmed for both −S9 and +S9. ^†^ Positive controls for −S9 are as follows: AF-2, 0.01 μg/plate for WP*uvrA*, TA100, and TA98; NaN_3_, 0.5 μg/plate for TA1535; 9-AA, 80 μg/plate for TA1537. ^‡^ All positive controls for +S9 are 2-AA with the following doses: 10 μg/plate for WP*uvrA*; 1.0 μg/plate for TA100; 2.0 μg/plate for TA1535; 0.5 μg/plate for TA98; 2.0 μg/plate for TA1537.

**Table 5:** Results of the bacterial reverse mutation test for *Enterococcus casseliflavus* KB1733 ^1^.

Dose (μg/Plate)	Mean Revertant Colonies per Plate									
	*Escherichia coli*		*Salmonella typhimurium*							
	WP2*uvrA*		TA100		TA1535		TA98		TA1537	
	−S9	+S9	−S9	+S9	−S9	+S9	−S9	+S9	−S9	+S9
Negative control *	29	25	121	131	9	14	22	29	6	12
156	30	22	115	131	13	10	23	30	10	15
313	24	26	125	140	7	9	23	28	7	11
625	24	20	124	133	11	10	20	34	4	12
1250	24	29	113	136	7	12	22	31	6	14
2500^+^	24	33	104	149	9	12	26	29	7	12
5000^+^	28	22	140	154	12	8	18	30	10	16
Positive control ^†,‡^	107	798	550	998	622	311	613	391	242	122

^1^ Abbreviations: −S9, in absence of S9; +S9, in presence of S9; 2500^+^ and 5000^+^, precipitation of the test article was observed at 2500 and 5000 μg/plate, respectively. * Water for injection was used as a negative control. ^+^ Precipitation of the test article was confirmed for both −S9 and +S9. ^†^ Positive controls for −S9 are as follows: AF-2, 0.01 μg/plate for WP*uvrA*, TA100, and TA98; NaN_3_, 0.5 μg/plate for TA1535; 9-AA, 80 μg/plate for TA1537. ^‡^ All positive controls for +S9 are 2-AA with the following doses: 10 μg/plate for WP*uvrA*; 1.0 μg/plate for TA100; 2.0 μg/plate for TA1535; 0.5 μg/plate for TA98; 2.0 μg/plate for TA1537.

## Data Availability

The datasets presented in this article are not readily available because they will be made available upon request pending approval of an application for data use and execution of a data use agreement and/or material transfer agreement with KAGOME Co., Ltd. Request to access the datasets should be directed to Shohei Satomi.

## Supplementary Materials

The following supporting information can be downloaded at: https://www.mdpi.com/article/10.3390/microorganisms12050953/s1, Table S1: The regions of the possible horizontal gene transfers in the chromosome of *Enterococcus casseliflavus* KB1733; Table S2: Individual animal weight at day 0, day 7 and day 14.
