# Supplementary material for: Identification and Safety Assessment of Enterococcus casseliflavus KB1733 Isolated from Traditional Japanese Pickle Based on Whole-Genome Sequencing Analysis and Preclinical Toxicity Studies
Source: Microorganisms. 2024 May 8;12(5):953. doi: 10.3390/microorganisms12050953 (PMC11123836; doi:10.3390/microorganisms12050953)
Supplement: Supplementary file 1 [file microorganisms-12-00953-s001.zip › microorganisms-2981761-supplementary.pdf]

Satomi S. *et al*., Identification and safety assessment of *Enterococcus casseliflavus* KB1733 isolated from traditional Japanese pickle based on whole-genome sequencing analysis and preclinical toxicity studies: Supplementary Files.

Supplementary Table S1. The regions of the possible horizontal gene transfers in the chromosome of *Enterococcus casseliflavus* KB1733 [1].

No	Start	Stop	Score	Threshold	No	Start	Stop	Score	Threshold
1	100,000	105,000	0.18	14.762	20	1,945,000	1,952,500	0.285	14.762
2	112,500	117,500	0.217	14.762	21	1,957,500	1,987,500	0.636	14.762
3	130,000	135,000	0.161	14.762	22	2,327,500	2,347,500	0.38	14.762
4	272,500	277,500	0.261	14.762	23	2,462,500	2,467,500	0.162	14.762
5	280,000	295,000	0.197	14.762	24	2,485,000	2,495,000	0.35	14.762
6	532,500	542,500	0.296	14.762	25	2,512,500	2,530,000	0.266	14.762
7	567,500	575,000	0.219	14.762	26	2,532,500	2,550,000	0.256	14.762
8	670,000	685,000	0.382	14.762	27	2,740,000	2,745,000	0.167	14.762
9	765,000	802,500	0.371	14.762	28	3,230,000	3,242,500	0.353	14.762
10	812,500	817,500	0.158	14.762	29	3,297,500	3,302,500	0.198	14.762
11	835,000	842,500	0.237	14.762	30	3,327,500	3,350,000	0.337	14.762
12	1,022,500	1,030,000	0.168	14.762	31	3,365,000	3,370,000	0.183	14.762
13	1,105,000	1,115,000	0.159	14.762	32	3,372,500	3,377,500	0.167	14.762
14	1,167,500	1,172,500	0.24	14.762	33	3,385,000	3,395,000	0.392	14.762
15	1,247,500	1,267,500	0.409	14.762	34	3,397,500	3,405,000	0.201	14.762
16	1,520,000	1,527,500	0.251	14.762	35	3,412,500	3,420,000	0.205	14.762
17	1,685,000	1,707,500	0.21	14.762	36	3,437,500	3,445,000	0.171	14.762
18	1,712,500	1,737,500	0.234	14.762	37	3,475,000	3,485,000	0.337	14.762
19	1,740,000	1,745,000	0.215	14.762	38	3,502,500	3,510,000	0.269	14.762

[1] The contigs in the chromosome of *E. casseliflavus* KB1733 with a score below or above the threshold are provided by Alien Hunter (version 1.1.0).

Supplementary Table S2. Individual animal weight at day 0, day 7 and day 14.[1]

	Animal weight (g)		
	Day 0	Day 7	Day 14
M 1	184	267	323
M 2	190	283	346
M 3	185	262	316
M 4	184	261	316
M 5	195	289	351
F 1	156	202	242
F 2	147	193	222
F 3	150	190	214
F 4	153	191	222
F 5	152	200	235

[1] Abbreviations: M1–M5, males; F1–F5, females.
